# The Influence of Immunization Route, Tissue Microenvironment, and Cytokine Cell Milieu on HIV-Specific CD8^+^ T Cells Measured Using Fluidigm Dynamic Arrays

**DOI:** 10.1371/journal.pone.0126487

**Published:** 2015-05-06

**Authors:** Shubhanshi Trivedi, Charani Ranasinghe

**Affiliations:** Molecular Mucosal Vaccine Immunology Group, Department of Immunology, The John Curtin School of Medical Research, The Australian National University, Canberra ACT, Australia; Harvard Medical School, UNITED STATES

## Abstract

Thirty different genes including cytokines, chemokines, granzymes, perforin and specifically integrins were evaluated in Peyer's patch-KdGag_197–205_-specific CD8+ T cells (pools of 100 cells) using Fluidigm 48.48 Dynamic arrays following three different prime-boost immunization strategies. Data revealed that the route of prime or the booster immunization differentially influenced the integrin expression profile on gut KdGag_197–205_-specific CD8+ T cells. Specifically, elevated numbers of integrin αE and αD expressing gut KdGag_197–205_-specific CD8+ T cells were detected following mucosal but not systemic priming. Also, αE/β7 and αD/β2 heterodimerization were more noticeable in an intranasal (i.n.)/i.n. vaccination setting compared to i.n./intramuscular (i.m) or i.m./i.m. vaccinations. Moreover, in all vaccine groups tested α4 appeared to heterodimerize more closely with β7 then β1. Also MIP-1β, RANTES, CCR5, perforin and integrin α4 bio-markers were significantly elevated in i.n./i.m. and i.m./i.m. immunization groups compared to purely mucosal i.n./i.n. delivery. Furthermore, when wild type (WT) BALB/c and IL-13 knockout (KO) mice were immunized using i.n./i.m. strategy, MIP-1α, MIP-1β, RANTES, integrins α4, β1 and β7 mRNA expression levels were found to be significantly different, in mucosal verses systemic KdGag_197–205_-specific CD8+ T cells. Interestingly, the numbers of gut KdGag_197–205_-specific CD8+ T cells expressing gut-homing markers α4β7 and CCR9 protein were also significantly elevated in IL-13 KO compared to WT control. Collectively, our findings further corroborate that the route of vaccine delivery, tissue microenvironment and IL-13 depleted cytokine milieu can significantly alter the antigen-specific CD8+ T cell gene expression profiles and in turn modulate their functional avidities as well as homing capabilities.

## Introduction

It is now well established that route of vaccine delivery can greatly influence the quality of HIV-specific CD8^+^ T cell immunity. Purely systemic immunization strategies intramuscular (i.m.) or intravenous (i.v.) immunizations) generate mainly long-lasting systemic immunity, whereas mucosal immunization (i.n, intrarectal (i.r.) or oral) is able to induce “long lasting” mucosal immune responses at the local and distant mucosa [1–4]. It has been shown that macaques vaccinated with i.m. DNA-HIV vaccine priming followed by i.n. or i.r. fowlpox virus (FPV)-HIV booster immunization generated enhanced local T-cell immunity in cervico-vaginal tissues, and they were protected against a mucosal SHIV challenge [5]. Studies where i.m. pDNA-HIV/ i.n FPV-HIV; i.n. FPV-HIV/ i.m. attenuated vaccinia virus (VV)-HIV and i.n. VV-HIV/ i.m. FPV-HIV were evaluated, i.n. FPV-HIV prime followed by i.m. VV-HIV booster vaccination has shown to induce robust polyfunctional HIV-specific T cell immunity compared to the two other prime-boost vaccination strategies [6]. Notably, control of HIV replication in elite controllers (< 1%) have been associated with enhanced high avidity polyfunctional HIV-specific CD8^+^ T cells [7, 8]. Our studies also clearly demonstrated that rFPV was an excellent mucosal delivery vector. We have also shown that compared to purely systemic (i.m FPV-HIV/ i.m VV-HIV) immunization regime, i.n. FPV-HIV prime followed by i.m VV-HIV booster immunization strategy induce robust long lasting CD8^+^ systemic and mucosal T cell responses to HIV-1 vaccine antigens [9], which were also of higher avidity [10, 11]. Furthermore, purely mucosal immunization i.n./i.n. or combined mucosal systemic i.n./i.m immunization induced HIV-specific CD8^+^ T cells with lower IL-4 and IL-13 expression compared to systemic immunization (i.m./i.m.), which were of higher avidity. Later studies using IL-13 KO mice have confirmed that IL-13 can significantly dampen the induction of effector and memory CD8^+^ T cells of higher avidity following vaccination [10, 12].

Studies have also shown that tissue microenvironment (*i*.*e*. mucosal vs. systemic compartments) influence the nature of immune responses induced at mucosal and systemic sites [13–15]. Unlike systemic compartment, due to the ability of antigens to be processed and presented via the microfold or M cells, immune responses induced in the mucosal compartment is uniquely different [16, 17]. It is known that, locally activated mucosal DCs but not splenic-DCs have the ability to imprint T cells with mucosal homing markers [18, 19]. For example, DCs purified from Peyer’s patches and mesenteric lymph nodes (MLNs) but not from spleen or peripheral lymph nodes induce T cell expression of both α_4_β_7_ and CCR9 *in vitro* and licence effector/memory T cells to home preferentially to intestinal epithelium [18, 19]. Notably, only gut-DCs (but not DCs from other lymphoid organs) can produce retinoic acid from retinol (vitamin A), and it has been shown that retinoic acid plays a critical role in imprinting of gut-homing specificities on T cells [20]. These studies highlights the importance of mucosal vaccination in the process of inducing long-lived mucosal-specific T cell immunity. Unfortunately, no clear biomarkers are currently available to measure HIV-specific mucosal vaccine-specific CD8^+^ T cell immunity, during human or NHP HIV vaccine trials, obtaining enough HIV-specific CD8 T cells from gut or rectal biopsies to evaluate mucosal immunity can be a difficult task. Specifically, due to small sample size obtaining meaningful data from FACS ELISPOT, can be a difficult task [21]. Thus, finding new biomarkers that can be used to evaluate mucosal immunity, under these circumstances can to be of great importance, and this forms the basis of this project.

Therefore, in this study, with the hope of identifying gene expression profiles unique to mucosal and systemic vaccination, thirty genes comprised of cytokines, chemokines, granzymes, perforin, markers which are involved in immune regulation and integrins (Table 1), were evaluated using Fluidigm 48.48 Dynamic array system. Specifically the array of integrins were selected as i) some of these molecules have shown to provoke lymphocyte homing to specific tissues, and ii) enhanced expression of certain homing receptors on antigen-specific T cells following mucosal vaccination has been associated with mucosal T cell homing and control of viral replication at the mucosae [22–24]. In this study mRNA expression profiles were evaluated in pools of hundred gut K^d^Gag_197–205_-specific (tetramer-specific) CD8^+^ T cells obtained from i.n./i.n. (purely mucosal), i.n./i.m. (mucosal/systemic) and i.m./i.m. (purely systemic) FPV-HIV/VV-HIV prime-boost vaccinated BALB/c mice and hundred gut and splenic K^d^Gag_197–205_-specific CD8^+^ T cells obtained from BALB/c and IL-13 KO animals following i.n./i.m. immunization. In our previous studies mucosal immunization [10], and IL-13 depleted cell milieu has shown to promote the induction of higher avidity HIV-specific CD8^+^ T cells, where avidity profile of IL-13 KO > WT BALB/C mice [12].

**Table 1:** Genes of interest used in this study for mRNA quantification using Fluidigm 48.48 dynamic array nanofluidic chip.

N	Gene symbol	Gene name	Category	Reference sequence	Assay ID
1	RpL32	Ribosomal protein L32	Endogenous standards	NM_172086.2	Mm02528467_g1
2	IFN-γ	Interferon gamma	Cytokines	NM_008337.3	Mm01168134_m1*
3	IL-2	Interleukin 2	Cytokines	NM_008366.2	Mm00434256_m1*
4	TNF-α	Tumor necrosis factor alpha	Cytokines	NM_013693.2	Mm00443258_m1*
5	IL-4	Interleukin-4	Cytokines	NM_021283.2	Mm00445259_m1*
6	IL-13	Interleukin-13	Cytokines	NM_008355.3	Mm00434204_m1*
7	IL-17	Interleukin-17	Cytokines	NM_010552.3	Mm00439618_m1*
8	MIP-1α (CCL3)	chemokine (C-C motif) ligand 3	Chemokines	NM_011337.2	Mm00441258_m1*
9	MIP-1β (CCL4)	chemokine (C-C motif) ligand 4	Chemokines	NM_013652.2	Mm00443111_m1*
10	RANTES (CCL5)	chemokine (C-C motif) ligand 5	Chemokines	NM_013653.3	Mm01302428_m1*
11	Gzm A	Granzyme A	Protease	NM_010370.2	Mm00439191_m1*
12	Gzm B	Granzyme B	Protease	NM_013542.2	Mm00442834_m1*
13	Gzm C	Granzyme C	Protease	NM_010371.2	Mm01313651_m1*
14	Prf1	Perforin 1	Defense protein	NM_011073.3	Mm00812512_m1*
15	Sell (CD62L)	L-selectin	Cell adhesion molecule	NM_011346.1	Mm00441291_m1*
16	Selplg (Psgl1)	P-selectin ligand	Receptor	NM_009151.3	Mm01204601_m1*
17	CD69	CD69 antigen	Receptor	NM_001033122.3	Mm01183378_m1*
18	CCR5	Chemokine (C-C motif) receptor 5	Receptor	NM_009917.5	Mm01216171_m1*
19	CCR7	Chemokine (C-C motif) receptor 7	Receptor	NM_007719.2	Mm01301785_m1*
20	CCR9	Chemokine (C-C motif) receptor 9	Receptor	NM_009913.5	Mm02620030_s1*
21	CCR10	Chemokine (C-C motif) receptor 10	Receptor	NM_007721.4	Mm01292449_m1*
22	Itgα4	Integrin alpha 4	Integrin	NM_010576.3	Mm00439770_m1*
23	ItgαE	Integrin alpha E	Integrin	2 Refseqs	Mm00434443_m1*
24	ItgαL	Integrin alpha L	Integrin	4 Refseqs	Mm00801807_m1*
25	ItgαM (CD11b)	Integrin alpha M	Integrin	2 Refseqs	Mm00434455_m1*
26	ItgαX (CD11c)	Integrin alpha X	Integrin	NM_021334.2	Mm00498698_m1*
27	ItgαD (CD11d)	Integrin alpha D	Integrin	NM_001029872.2	Mm01159115_m1*
28	Itgβ1	Integrin beta 1 (fibronectin receptor beta)	Integrin	NM_010578.2	Mm01253228_g1*
29	Itgβ2	Integrin beta 2	Integrin	NM_008404.4	Mm00434513_m1*
30	Itgβ7	Integrin beta 7	Integrin	NM_013566.2	Mm00442916_m1*

Note: In assay ID column * indicates the relevant accession numbers of the TaqMan primer probe sets used in the study obtained from Applied Biosystems (Life Technologies, USA).

## Results

### Mucosal and systemic vaccination can induce distinct mRNA expression profiles in gut-K^d^Gag_197–205_-specific CD8^+^ T cells

**Fig 1:**
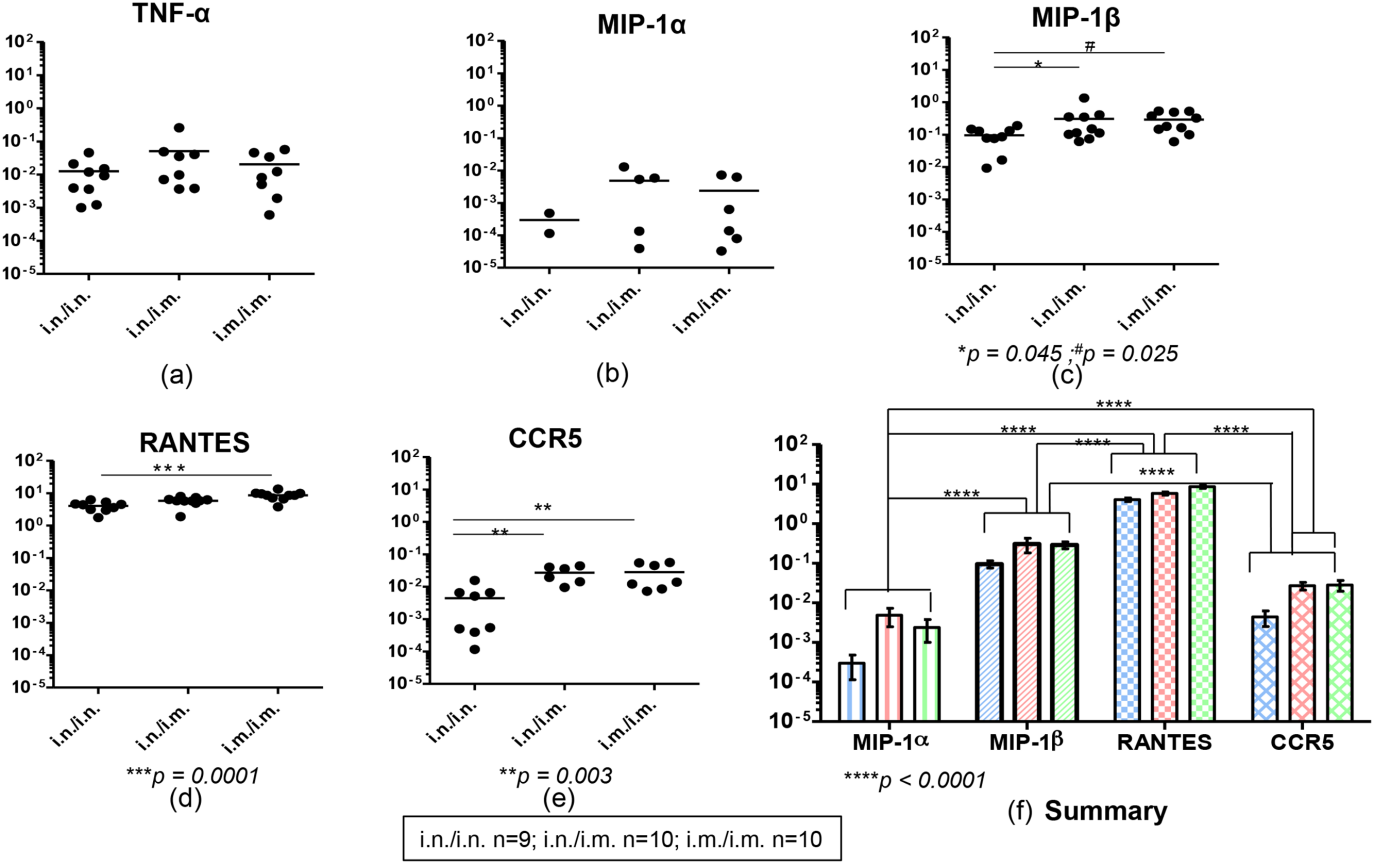
Evaluation of TNF-α, chemokines and chemokine receptor mRNA expression in gut K^d^Gag_197–205_–specific CD8^+^ T cells following i.n./i.n., i.n./i.m. and i.m./i.m. vaccination. BALB/c mice (n = 4–5 per group) were primed i.n. or i.m. with FPV-HIV and boosted i.n. or i.m. with VV-HIV, fourteen days post boost immunization Peyer’s patches were pooled from all animals and cells were then stained with K^d^Gag_197–205_ tetramer. These gut K^d^Gag_197–205—_specific CD8^+^ T cells were sorted in hundred cell pools (i.n./i.n. n = 9, i.n./i.m. n = 10 and i.m./i.m. n = 10 hundred cell pools) in a 96-wells plate, cDNA was synthesized and mRNA expression profiles were evaluated using Fluidigm 48.48 dynamic arrays as described in methods. The log mRNA expression normalised to housekeeping gene L32 (2^-ΔCT^, where ΔCT = CT_gene_—CT_L32_) of TNF-α (a), MIP-1α (b), MIP-1β (c), RANTES (d), CCR5 (e) is shown. All chemokines tested and CCR5 expression in gut K^d^Gag_197–205_-specific CD8^+^ T cells following i.n./i.n. (blue), i.n./i.m. (red) and i.m./i.m.(green) vaccination is summarized (f). In each plot (a-e), black circles indicate a pool of hundred gut K^d^Gag_197–205_-specific CD8^+^ T cells and black line indicate mean expression. The experiment was repeated three times. Data represent the three experiments.

WT BALB/c (n = 4–5) were primed with FPV-HIV and boosted with VV-HIV vaccine using i.n./i.n., i.n./i.m. or i.m./i.m. vaccine regimes as indicated in methods, fourteen days post booster immunization gut K^d^Gag_197–205_-specific CD8^+^ T cell were sorted in groups of hundred cells and mRNA expression profiles of 30 genes of interest (Table 1) were evaluated using Fluidigm 48.48 Dynamic arrays. When expression of cytokines IFN-γ, TNF-α, IL-2, IL-4, IL-13 and IL-17 mRNA by gut K^d^Gag_197–205_-specific CD8^+^ T cells were evaluated, although no IFN-γ, IL-2, IL-4, IL-13 and IL-17 mRNA were detected, majority of the pools of hundred gut K^d^Gag_197–205_-specific CD8^+^ T cells obtained from the three immunization regimes (i.n./i.n.- 9 out of 9; i.n./i.m. and i.m./i.m.- 8 out of 10, *i*.*e*. 80–100%) expressed TNF-α, and the expression profile between the three regimes were very similar (Fig 1a).

When chemokines MIP-1α, MIP-1β and RANTES mRNA expression profiles were evaluated, data indicated that although the level of MIP-1α mRNA expression was similar between the three regimes, the number of hundred gut K^d^Gag_197–205_-specific CD8^+^ T pools expressing MIP-1α were higher in the i.n./i.m and i.m./i.m. regimes compared to the purely mucosal i.n./i.n. prime-booster regime (Fig 1b). Data also revealed that all cell pools (i.n./i.n.- 9/9, i.n./i.m and i.m./i.m.-10/10) expressed MIP-1β and RANTES mRNA and the expression level of MIP-1β mRNA was significantly higher in the i.n./i.m and i.m./i.m. groups and the RANTES mRNA in the i.m./i.m. group compared to the i.n./i.n. group (MIP-1β i.n./i.n. vs. i.n./i.m. *p* = 0.045 and i.n./i.n. vs. i.m./i.m. *p* = 0.025; RANTES i.n./i.n. vs i.m./i.m. *p* = 0.0001) (Fig 1c and 1d). The level of CCR5 mRNA expression by these gut K^d^Gag_197–205_-specific CD8^+^ cells were also found to be similar to that of MIP-1β where i.n./i.n. vs. i.n./i.m and i.n./i.n. vs i.m./i.m. were significantly elevated *p* = 0.003 (Fig 1e). In all three immunization regimes tested, the hierarchy of these chemokine and/or their receptor mRNA expression by gut-K^d^Gag_197–205_-specific CD8^+^ T cells was found to be RANTES > MIP-1β > CCR5 > MIP-1α (Fig 1f).

**Fig 2:**
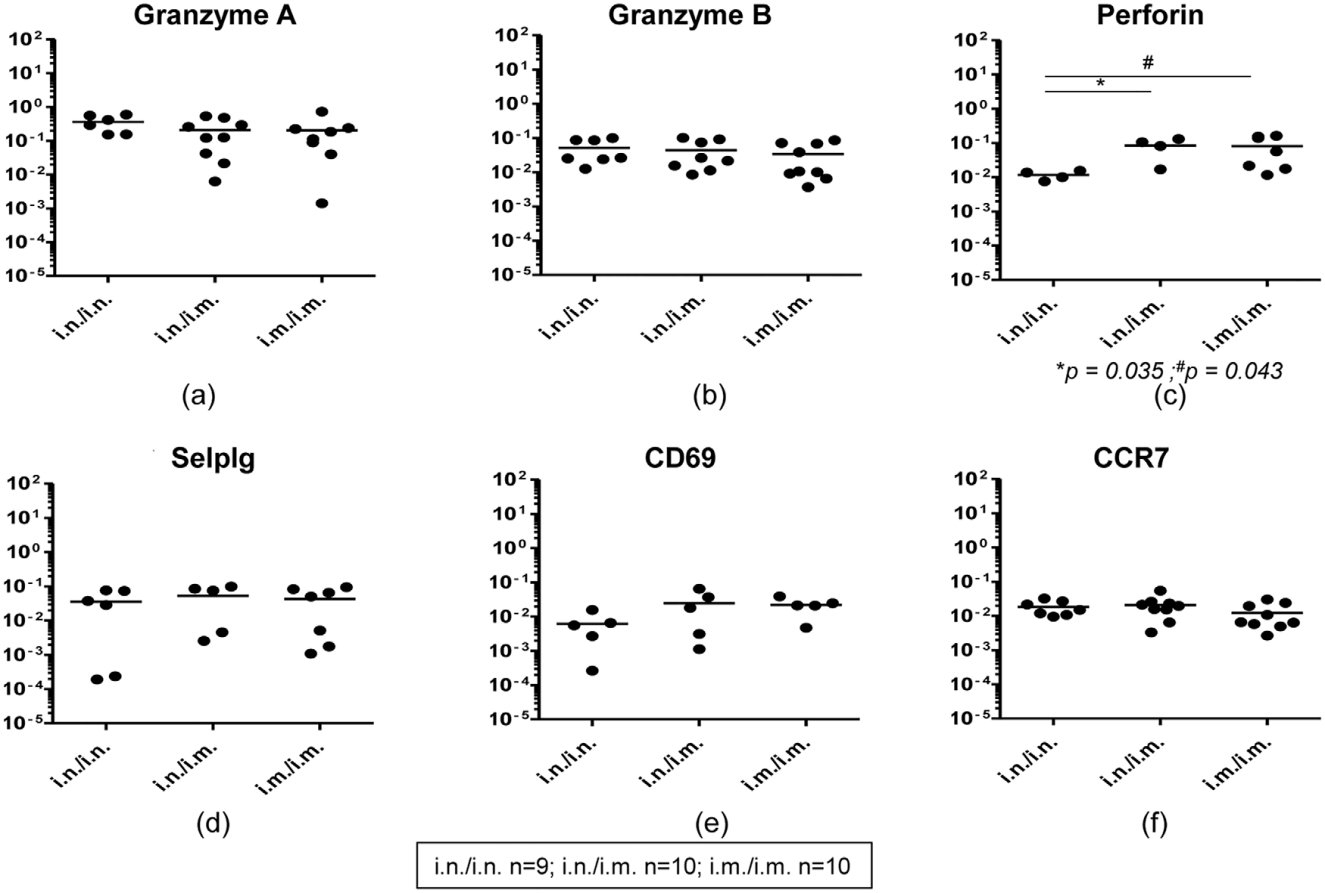
Evaluation of granzymes, perforin and receptor mRNA expression in gut K^d^Gag_197–205_–specific CD8^+^ T cells following i.n./i.n., i.n./i.m. and i.m./i.m. vaccination. mRNA expression profile in i.n./i.n., i.n./i.m. and i.m./i.m. immunized pools of hundred gut K^d^Gag_197–205_-specific CD8^+^ T cells (i.n./i.n. n = 9, i.n./i.m. n = 10 and i.m./i.m. n = 10 pools) were evaluated using Fluidigm 48.48 dynamic arrays as described in methods. The log mRNA expression normalised to housekeeping gene L32 (2^-ΔCT^) of granzyme-A (a), granzyme-B (b), perforin (c), selplg or P-selctin ligand (d), CD69 (e) and CCR9 (f) is shown. In each plot (a-f), black circles indicate a pool of hundred gut K^d^Gag_197–205_-specific CD8^+^ T cells and black line indicate mean expression. The experiment was repeated three times. Data represent the three experiments.

When the levels of granzymes (A,B and C) and perforin mRNA expression by gut- K^d^Gag_197–205_-specific CD8^+^ T cells were compared, in all three immunization regimes major proportion of the hundred cell pools were found to express granzyme A and B mRNAs (60–90%) but no granzyme C expression was detected in any of the immunization regimes tested. Also, the levels of granzyme A and B mRNA expression were found to be similar between the three immunization regimes tested (Fig 2a and 2b). In contrast, the number of perforin expressing gut-K^d^Gag_197–205_-specific CD8^+^ T cell pools were lower (40–60%), and the level of perforin expression was significantly higher in the i.n./i.m and i.m./i.m. regimes compared to i.n./i.n. strategy (i.n./i.n. vs. i.n./i.m. *p* = 0.035 and i.n./i.n. vs. i.m./i.m. *p* = 0.043) (Fig 2c).

Furthermore, when the expression of CD62L (also represented as ‘sell’), CD69, CCR7, CCR9, CCR10 and P-selectin ligand (selplg) mRNA by gut-K^d^Gag_197–205_-specific CD8^+^ T cells were evaluated between the three immunization regimes, no expression of CD62L, CCR9 and CCR10 were detected. However, compared to CCR7, lower numbers of gut-K^d^Gag_197–205_-specific CD8^+^ T cell pools were found to be positive for P-selectin ligand and CD69 and no significant differences in the expression levels of these mRNA were detected between three immunization regimes tested (Fig 2d–2f).

### Different routes of vaccine delivery can induce distinct integrin mRNA expression profiles in gut- K^d^Gag_197–205_-specific CD8^+^ T cells

**Fig 3:**
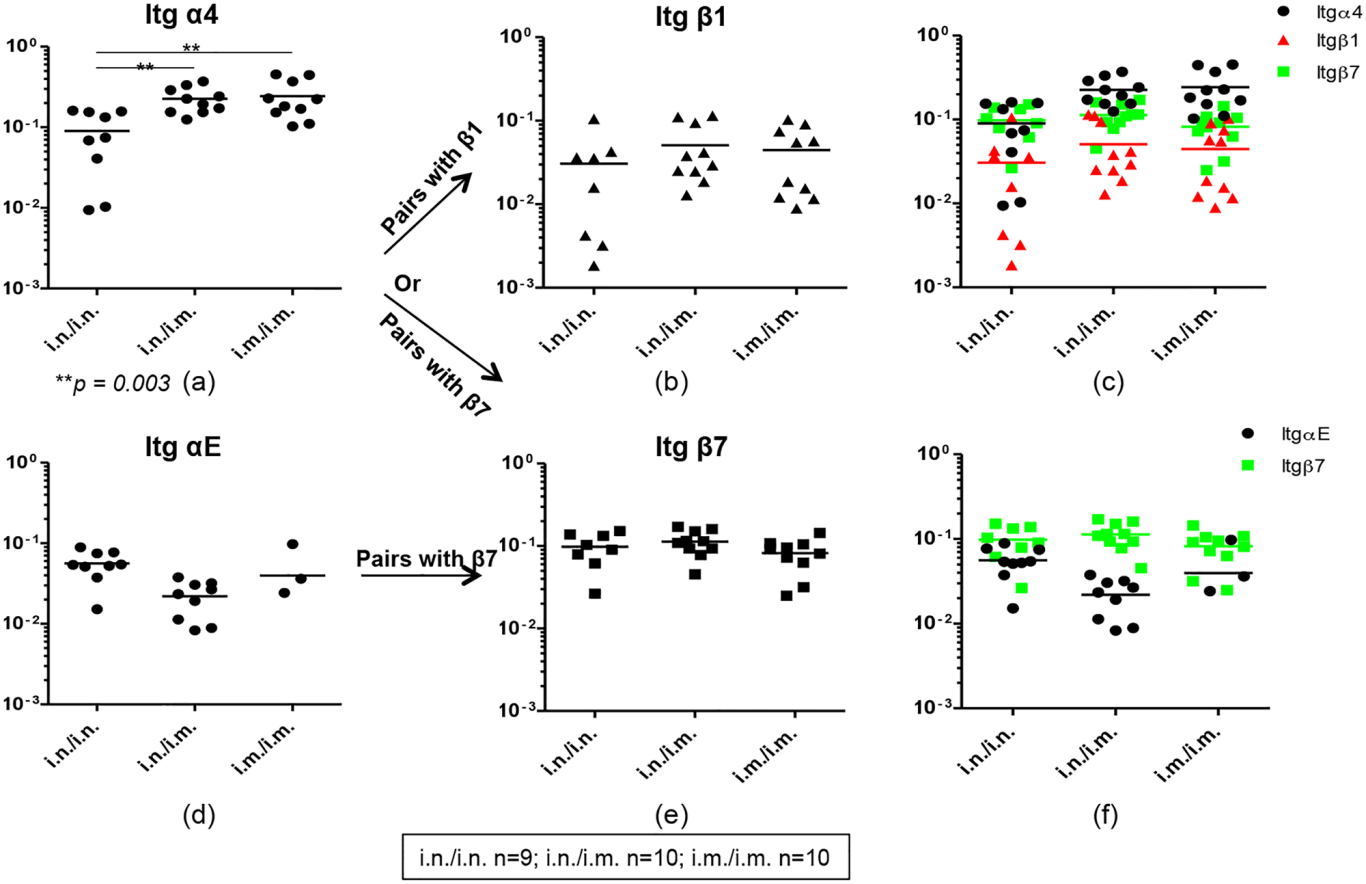
Evaluation of integrins α4, αE, β1 and β7 mRNA expression in gut K^d^Gag_197–205_-specific CD8^+^ T cells following i.n./i.n., i.n./i.m. and i.m./i.m. vaccination. Integrins mRNA expression profile in i.n./i.n., i.n./i.m. and i.m./i.m. immunized pools of hundred gut K^d^Gag_197–205_-specific CD8^+^ T cells (i.n./i.n. n = 9, i.n./i.m. n = 10 and i.m./i.m. n = 10 pools) were evaluated using Fluidigm 48.48 dynamic arrays as described in methods. The log mRNA expression normalised to housekeeping gene L32 of integrin α4 (a), β1 (b), α4 (black circle) which can pair up with β1 (red triangle) and β7 (green square) (c), αE (d), β7 (e) and αE (black circle) which can pair up with β7 (green square) (f) is shown. The *p* values were calculated using one-way ANOVA. In all the plots circles or triangles or square indicate a pool of hundred gut K^d^Gag_197–205_-specific CD8^+^ T cells and line indicate mean expression. The experiment was repeated three times. Data represent the three experiments.

It is established that α4 can heterodimerize with either β7 or β1 while αE can only heterodimerize with β7. In the three immunization regimes tested, all the pools of hundred gut-K^d^Gag_197–205_-specific CD8^+^ T cells (100%) were found to express integrin α4, and the expression was significantly higher in i.n./i.m. and i.m./i.m. compared to the purely mucosal i.n./i.n. immunization regime (i.n./i.n. vs. i.n./i.m. and i.n./i.n. vs. i.m./i.m. *p* = 0.003) (Fig 3a). Data revealed that 100% of gut-K^d^Gag_197–205_-specific CD8^+^ T cells were also positive for integrins β1 and β7 mRNA in all the groups tested (Fig 3b & 3e). The expression level hierarchy of β1 mRNA was i.n./i.m > i.m./i.m. > i.n./i.n. whereas expression level of β7 mRNA was similar in all the groups (Fig 3b & 3e). Interestingly, when the α4, β1 and β7 mRNA expression by gut-K^d^Gag_197–205_-specific CD8^+^ T cells were compared between the three immunization regimes (Fig 3c), α4 and β7 expression profiles were found to be relatively higher and closely linked specifically in i.n./i.n. and i.n./i.m. groups compared to the mean expression level of α4 and β1 mRNAs in all the groups (Fig 3c).

Although the levels of integrin αE (which can also pair with β7 integrin) mRNA expression were similar between the three immunization regimes, 90–100% (i.n./i.n- 9/9, i.n./i.m.- 9/10) of the pools of hundred gut-K^d^Gag_197–205_-specific CD8^+^ T cell obtained from i.n./i.n. and i.n./i.m regimes (mucosally prime groups) expressed integrin αE compared to the purely systemic immunization strategy (only 3/10 or 30% of hundred cell pools expressed αE) (Fig 3d). Next when the αE and β7 mRNA expression by gut-K^d^Gag_197–205_-specific CD8^+^ T cells were compared between the three immunization regimes, αE and β7 expression profiles were found to be more closely associated in the i.n./i.n. purely mucosal vaccinated group compared to the i.n./i.m combined mucosal systemic or i.m./i.m. purely systemic vaccinated groups (Fig 3f).

**Fig 4:**
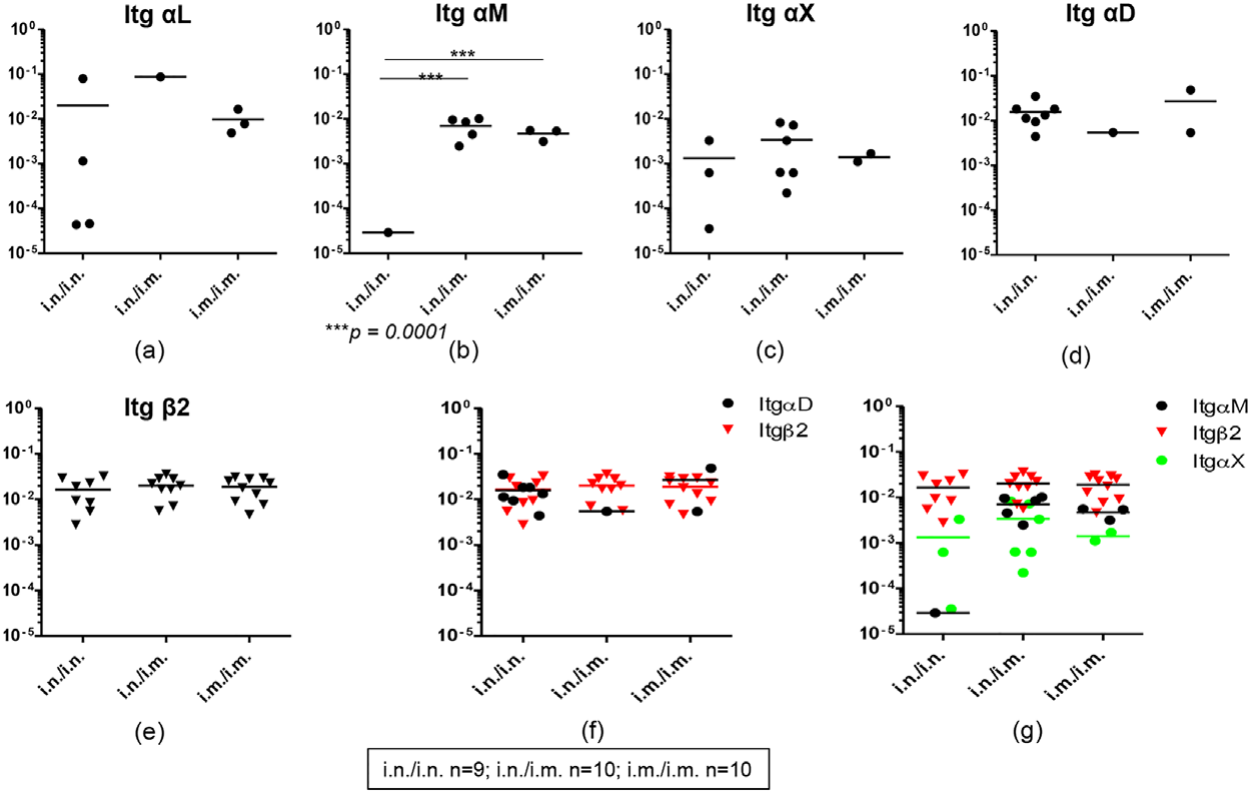
Evaluation of integrins αL, αM, αX, αD and β2 mRNA expression in gut K^d^Gag_197–205_–specific CD8^+^ T cells following i.n./i.n., i.n./i.m. and i.m./i.m. vaccination. The log mRNA expression normalised to housekeeping gene L32 of integrin αL (a), αM (b), αX (c), αD (d), β2 (e) αD (black circle) and β2 (inverted red triangle) (f) and αX (green circle), αM (black circle) which can pair up with β2 (inverted red triangle) (g) is shown. The *p* values were calculated using one-way ANOVA. In all the plots circles or triangles or square indicate a pool of hundred gut K^d^Gag_197–205_-specific CD8^+^ T cells and line indicate mean expression. The experiment was repeated three times. Data represent the three experiments.

Furthermore, integrins αL, αM, αX and αD which can heterodimerize with β2 integrin were evaluated in the three immunization regimes. Very low proportion of cell pools (1/10) were found to express integrin αL in i.n./i.m. group compared to the purely mucosal and purely systemic immunization groups, also the expression levels were not significantly different between the three groups tested (Fig 4a). Data indicated that higher numbers of hundred cell pools expressed integrin αM mRNA in i.n./i.m. group (~50%) and i.m./i.m. group (~30%) compared to i.n./i.n. group (~10%) (Fig 4b). Also, the expression was significantly higher in i.n./i.m and i.m./i.m. compared to purely mucosal i.n./i.n. regime (Fig 4b). Major proportion of the hundred cell pools (60%) obtained from i.n/i.m. group were found to express integrin αX, and the expression levels were also found to be relatively higher in this group compared to purely mucosal and purely systemic immunization regimes (Fig 4c). Unlike the expression pattern of integrins αM and αX positive gut-K^d^Gag_197–205_-specific CD8^+^ T cells where the expression profile hierarchy was i.n./i.m. > i.m./i.m. > i.n./i.n., higher proportions (80%) of gut-K^d^Gag_197–205_-specific CD8^+^ T cells expressed integrin αD mRNA in purely mucosal i.n./i.n. regime compared to i.n./i.m. (10%) and i.m./i.m. (20%) groups (Fig 4d). Interestingly, in all the immunization regimes tested, 90–100% of gut-K^d^Gag_197–205_-specific CD8^+^ T cells were found to express integrin β2 and also the expression levels were similar between the groups (Fig 4e). Next the integrin αD and β2 mRNA expression by gut-K^d^Gag_197–205_-specific CD8^+^ T cells were compared between the three immunization regimes (Fig 4f). Based on 80% of cells in the i.n./i.n. group expressing integrin αD and 100% of cells also expressing integrin β2, data suggest that integrin αD and β2 could be more closely associated with i.n. delivery compared to i.m. delivery (Fig 4f). Similarly, when integrin αM, αX and β2 were compared between the three groups (Fig 4g), in i.n./i.m. and i.m./i.m. groups the integrins αM and β2 were found to be relatively higher and closely associated compared to integrins αX and β2 (Fig 4g).

### WT BALB/c and IL-13 KO mice have distinct mRNA expression profiles in systemic and mucosal K^d^Gag_197–205_-specific CD8^+^ T cells

**Fig 5:**
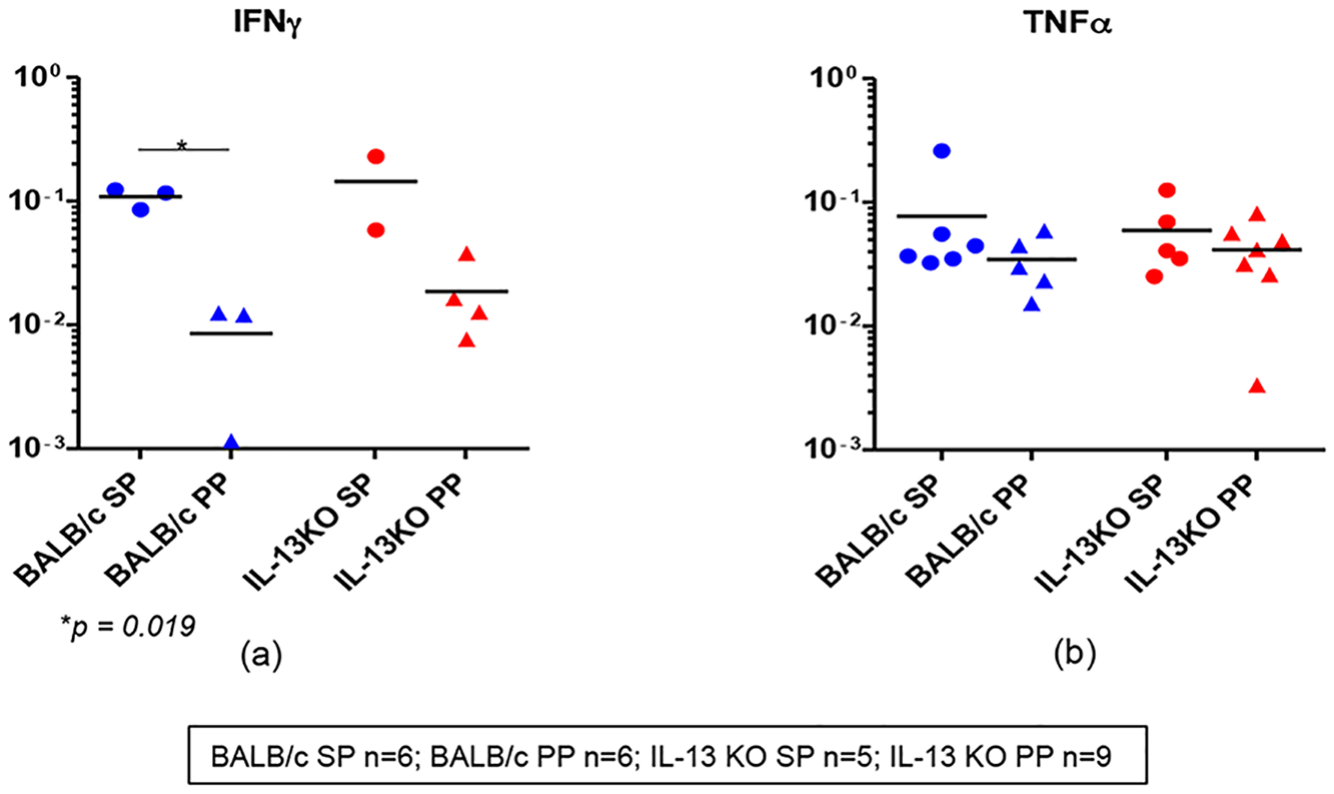
Evaluation of cytokine mRNA expression in WT BALB/c and IL-13 KO gut and splenic K^d^Gag_197–205_-specific CD8^+^ T cells following i.n./i.m. vaccination. WT BALB/c and IL-13 KO mice (n = 4–5/ group) were prime-boost immunized with i.n. FPV-HIV/ i.m. VV-HIV. Fourteen days post booster vaccination mRNA expression profiles in gut and splenic K^d^Gag_197–205_-specific CD8^+^ T cells were evaluated using Fluidigm 48.48 dynamic arrays as described in methods. WT BALB/c spleen (SP) and Peyer’s patch (PP) n = 6 pools of hundred K^d^Gag_197–205-_specific CD8^+^ T cells are indicated by blue circle and triangle respectively. IL-13 KO mice spleen n = 5 and Peyer’s patch n = 9 pools of hundred K^d^Gag_197–205_-specific CD8^+^ T cells are indicated by red circles and triangles respectively. Data indicate log mRNA expression of IFN-γ (a) and TNF-α (b) normalised to housekeeping gene L32. The experiment was repeated three times. Data represent the three experiments.

We have shown that i.n. FPV-HIV/ i.m. VV-HIV immunization strategy can induce robust and sustained high avidity systemic and mucosal CD8^+^ T cell responses [10]. Thus, in this part of the study, WT BALB/c and IL-13 KO mice (n = 4–5) were immunized i.n. FPV-HIV/ i.m. VV-HIV strategy, 14 days post booster vaccination, gut and splenic K^d^Gag_197–205_-specific CD8^+^ T cell were sorted in groups of hundred cells, and mRNA expression profiles of the above same 30 genes were evaluated using Fluidigm 48.48 Dynamic arrays (Table 1). Interestingly, no IL-2, IL-4, IL-13, IL-17 mRNA expression were detected in gut and splenic K^d^Gag_197–205_-specific CD8^+^ T cells in all mice groups tested. IFN-γ were detected in 40–50% of the hundred cell pools obtained from spleen (WT BALB/c - 3/6, IL-13 KO - 2/5) and Peyer’s patch (WT BALB/c - 2/6, IL-13 KO - 4/9) and the IFN-γ mRNA expression level was relatively higher in splenic K^d^Gag_197–205_-specific CD8^+^ T cells (WT spleen vs. Peyer’s patch *p* = 0.019) compared to Peyer’s patches (Fig 5a). Unlike IFN-γ mRNA expression, much greater proportion of cells were found to express TNF-α (WT BALB/c spleen - 6/6, Peyer’s patch - 5/6; and IL-13 KO spleen - 5/5, Peyer’s patch - 7/9), but no difference in the expression profiles were detected between gut and splenic K^d^Gag_197–205_-specific CD8^+^ T cells obtained from the two mice groups (Fig 5b).

**Fig 6:**
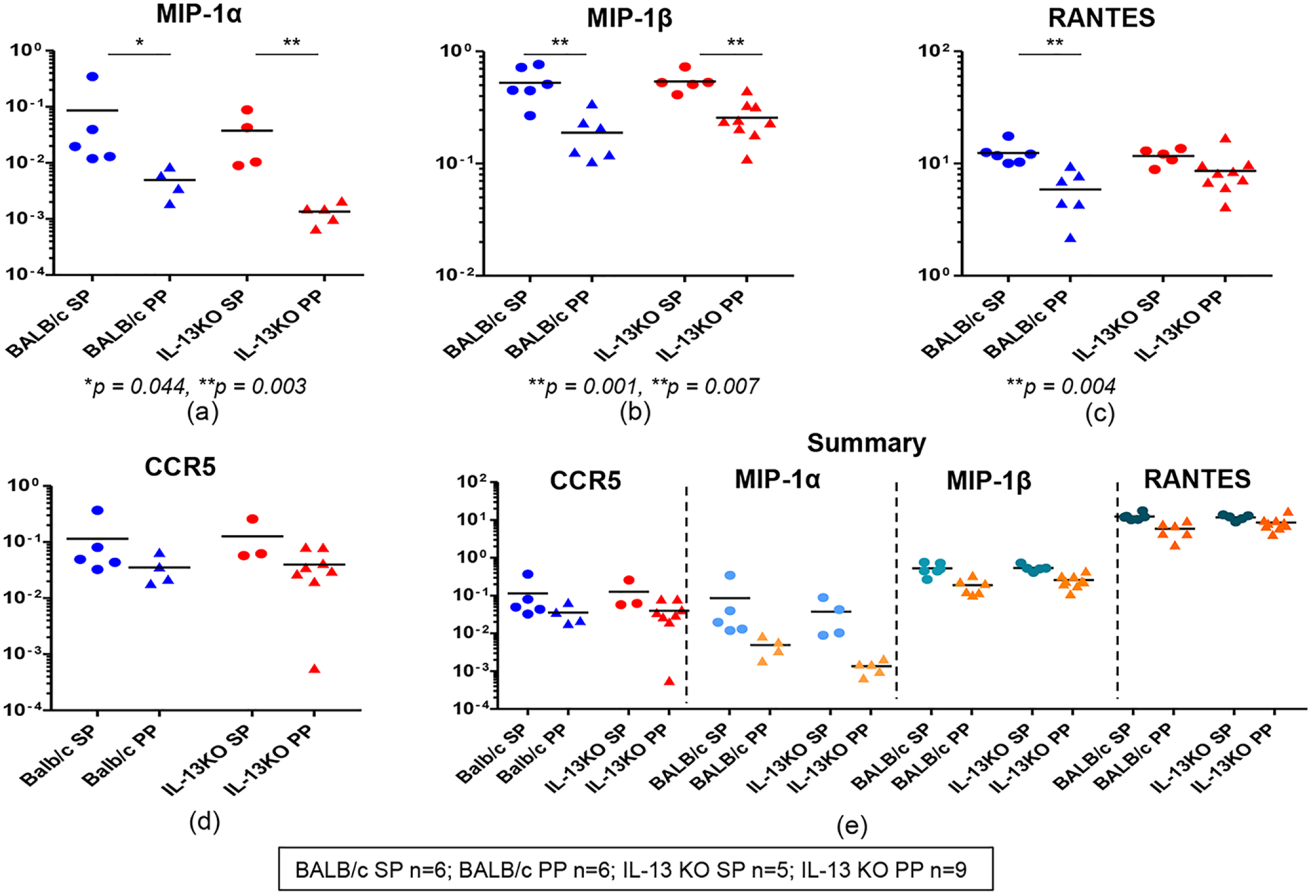
Evaluation of chemokines and chemokine receptor mRNA expression in WT BALB/c and IL-13 KO gut and splenic K^d^Gag_197–205_-specific CD8^+^ T cells following i.n./i.m. vaccination. **Exactly as in** fig 5 WT BALB/c and IL-13 KO mice (n = 4–5/ group) were prime-boost immunized with i.n. FPV-HIV/ i.m. VV-HIV. and mRNA expression profiles in gut and splenic K^d^Gag_197–205_-specific CD8^+^ T cells were evaluated. WT BALB/c spleen (SP) and Peyer’s patch (PP) n = 6 pools of hundred K^d^Gag_197–205_-specific CD8^+^ T cells are indicated by blue circle and triangle respectively. IL-13 KO mice spleen n = 5 and Peyer’s patch n = 9 pools of hundred K^d^Gag_197–205_-specific CD8^+^ T cells are indicated by red circles and triangles respectively. Data indicate log mRNA expression of MIP-1α (a) MIP-1β (b) and RANTES (c) CCR5 (d) normalised to housekeeping gene L32. Fig 6e indicates the summary of chemokine and CCR5 data.

When chemokines MIP-1α, MIP-1β and RANTES mRNA expression in WT and IL-13 KO gut and splenic K^d^Gag_197–205_-specific CD8^+^ T cells were evaluated, compared to MIP-1β and RANTES relatively lower numbers of gut and splenic-K^d^Gag_197–205_-specific CD8^+^ T cell pools expressed MIP-1α mRNA (WT BALB/c spleen - 5/6, Peyer’s patch - 4/6; and IL-13 KO spleen - 4/5, Peyer’s patch - 5/9) (Fig 6a). In both WT and IL-13 KO groups, the expression levels of MIP-1α were significantly elevated in splenic K^d^Gag_197–205_-specific CD8^+^ T cells compared to Peyer’s patches (spleen vs. Peyer’s patch *p* = 0.044 and 0.003 respectively) (Fig 6a). Similarly, in all the groups tested even though 100% of K^d^Gag_197–205_-specific CD8^+^ T cells were positive for MIP-1β and RANTES, the expression levels were significantly higher in spleen compared to Peyer’s patch (MIP-1β expression in WT and IL-13KO, spleen vs. Peyer’s patch *p* = 0.001 and 0.007 respectively; RANTES in WT group spleen vs. Peyer’s patch *p* = 0.004) (Fig 6b & 6c). Interestingly, in these groups the level of CCR5 mRNA expression between gut and splenic K^d^Gag_197–205_-specific CD8^+^ T cells were very similar, and compared to RANTES and MIP1- β, the proportion of cells expressing CCR5 were relatively lower (WT BALB/c spleen - 5/6, Peyer’s patch - 4/6; and IL-13 KO spleen - 3/5, Peyer’s patch - 8/9) (Fig 6d). In WT BALB/c or IL-13 KO group the hierarchy of chemokine and/or their receptor mRNA expression by gut and splenic K^d^Gag_197–205_-specific CD8^+^ T cells was found to be RANTES > MIP-1β > MIP-1α ≥ CCR5 (Fig 6e).

**Fig 7:**
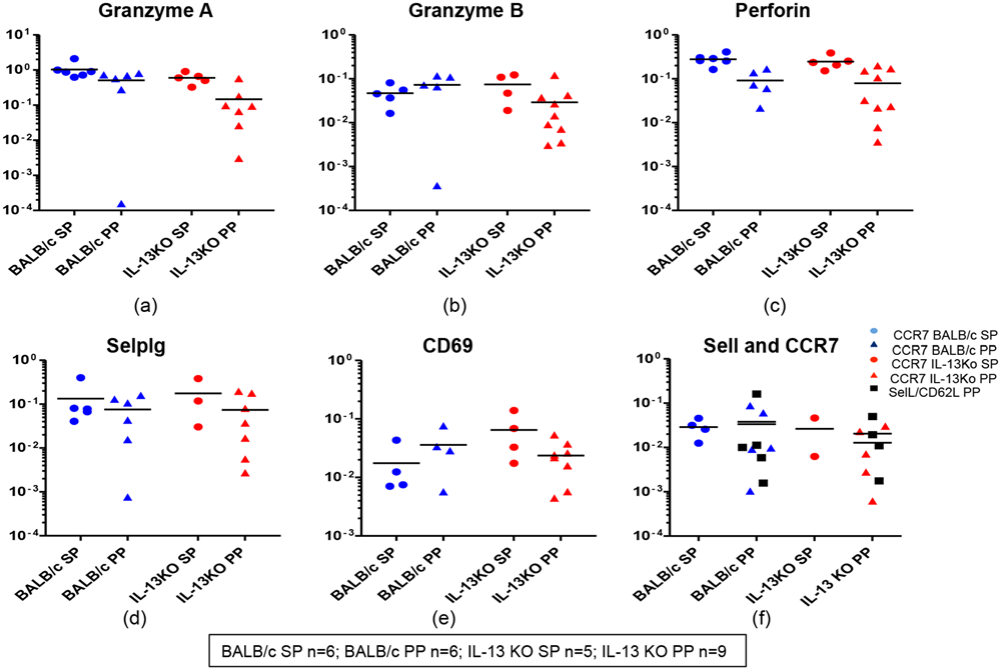
Evaluation of granzymes, perforin and receptor mRNA expression in WT BALB/c and IL-13 KO gut and splenic K^d^Gag_197–205_-specific CD8^+^ T cells following i.n./i.m. vaccination. mRNA expression profiles in WT BALB/c and IL-13KO gut and splenic K^d^Gag_197–205-_specific CD8^+^ T cells were evaluated using Fluidigm 48.48 dynamic arrays as described in methods. WT BALB/c spleen (SP) and Peyer’s patch (PP) n = 6 pools of hundred K^d^Gag_197–205-_specific CD8^+^ T cells are indicated by blue circle and triangle respectively. IL-13 KO mice spleen n = 5 and Peyer’s patch n = 9 pools of hundred K^d^Gag_197–205-_specific CD8^+^ T cells are indicated by red circles and triangles respectively. Data indicate log mRNA expression of granzyme A (a) granzyme B (b) perforin (c) selplg or P-selectin ligand (d) CD69 (e) and memory markers sell (CD62L) and CCR7 (f) normalised to housekeeping gene L32. The experiment was repeated three times. Data represent the three experiments.

When the expression of granzyme A, B and C mRNA in WT and IL-13 KO gut and splenic K^d^Gag_197–205_-specific CD8^+^ T cells were evaluated, major proportion of the hundred cell pools were found to express granzyme A and B mRNAs (80–100%) (Fig 7a & 7b) but no granzyme C expression was detected in any of the groups tested. Although the expression levels of granzyme A and B were found to be similar in WT and IL-13 KO splenic and gut K^d^Gag_197–205_-specific CD8^+^ T cells (Fig 7a & 7b) 100% (9/9) of the IL-13 KO gut-specific cell pools expressed B. Interestingly, in both compartments in WT and IL-13 KO groups 80–100% of hundred cell pools were positive for perforin, but expression levels were relatively higher in spleen compared to Peyer’s patch (Fig 7c). Furthermore, in WT and IL-13 KO groups, similar proportions of cells were found to express P-selectin ligand and CD69 mRNA in both compartments (WT BALB/c spleen- 4 to 5/6, Peyer’s patch- 4 to 6/6; and IL-13 KO spleen- 3 to 4/5, Peyer’s patch- 7 to 8/9), and their expression levels were also very similar (Fig 7d & 7e).

Interestingly, when the memory markers CD62L and CCR7 mRNA expression was evaluated in all the groups, the CD62L mRNA expression was only detected in gut K^d^Gag_197–205_-specific CD8^+^ (WT BALB/c 5/6 and IL-13 KO 4/9) whereas CCR7 mRNA expression was detected in both splenic and gut K^d^Gag_197–205_-specific CD8^+^ T cells (WT BALB/c spleen- 4/6, Peyer’s patch- 5/6; and IL-13 KO spleen- 2/5, Peyer’s patch- 5/9) (Fig 7f). The expression levels of CCR7 mRNA were found to be similar in the two compartments (Fig 7f). However, CD62L and CCR7 were found to be closely associated in Peyer’s Patch samples compared to spleen (Fig 7f).

### WT and IL-13 KO mice have distinct integrins and CCR9 mRNA expression profiles in gut and splenic K^d^Gag_197–205_-specific CD8^+^ T cells

**Fig 8:**
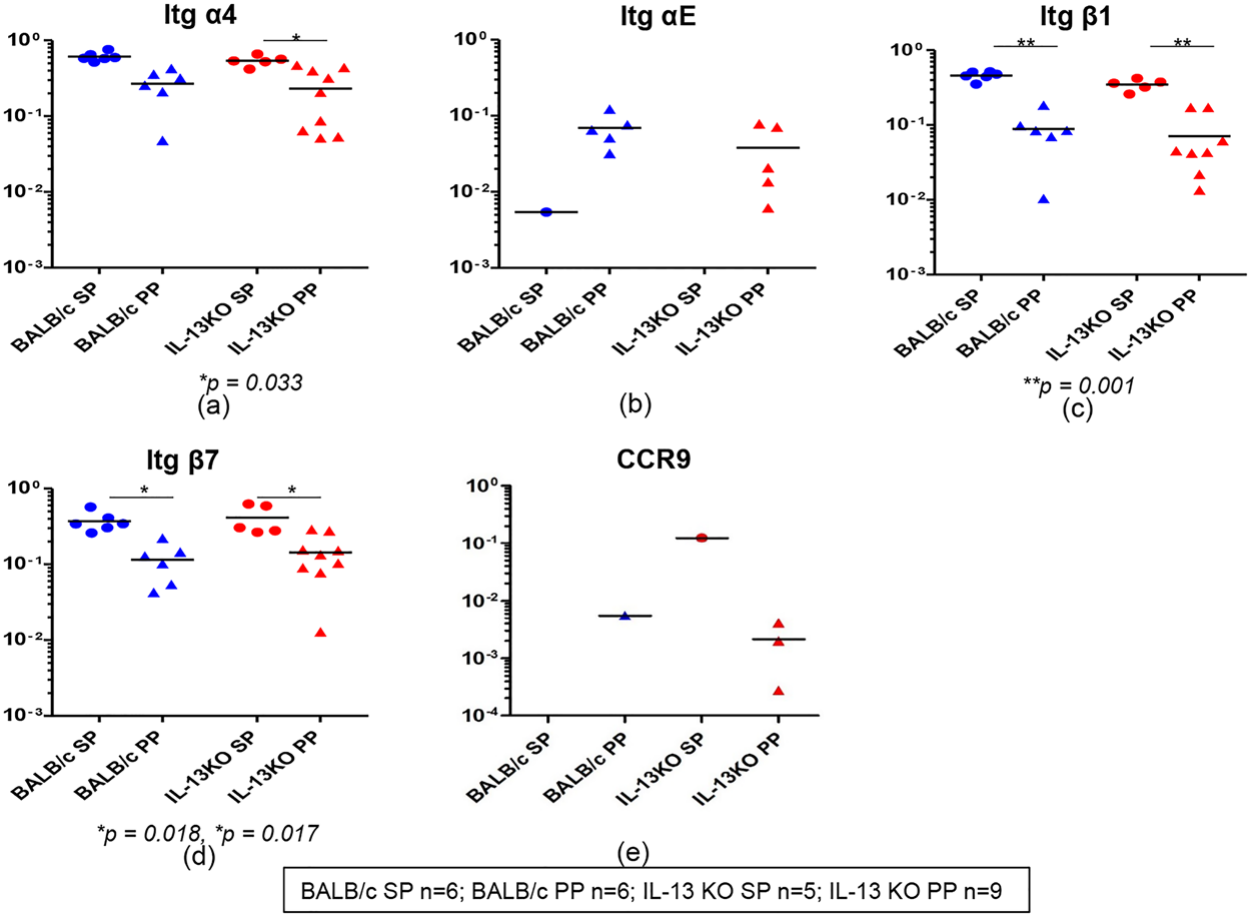
Evaluation of integrins α4, αE, β1and β7 and CCR9 mRNA expression in WT BALB/c and IL-13 KO gut and splenic K^d^Gag_197–205_-specific CD8^+^ T cells following i.n./i.m. vaccination. mRNA expression profiles in WT BALB/c and IL-13KO gut and splenic K^d^Gag_197–205-_specific CD8^+^ T cells were evaluated using Fluidigm 48.48 dynamic arrays as described in methods. WT BALB/c spleen (SP) and Peyer’s patch (PP) n = 6 pools of hundred K^d^Gag_197–205-_specific CD8^+^ T cells are indicated by blue circle and triangle respectively. IL-13 KO mice spleen n = 5 and Peyer’s patch n = 9 pools of hundred K^d^Gag_197–205-_specific CD8^+^ T cells are indicated by red circles and triangles respectively. Data indicate log mRNA expression of integrin α4 (a) αE (b) β1 (c) β7 (d) and CCR9 (e) normalised to housekeeping gene L32. The experiment was repeated three times. Data represent the three experiments.

Integrins α4, αE, β1, β7 and CCR9 expression were evaluated in WT BALB/c and IL-13 KO mice gut and splenic K^d^Gag_197–205_-specific CD8^+^ T cells. Data revealed that all gut and splenic cells expressed integrin α4 mRNA and expression levels were relatively higher in spleen compared to Peyer’s patches, specifically in IL-13 KO mice where spleen vs. Peyer’s patch *p* = 0.033 (Fig 8a). In contrast, in both the groups tested, majority of hundred cell pools that expressed integrin αE were detected in Peyer’s patch (WT BALB/c ~80%, IL-13 KO ~55%) but not in spleen and expression levels were also found to be elevated in gut-K^d^Gag_197–205_-specific CD8^+^ T cells (Fig 8b). Data also revealed all the cell pools expressed integrins β1 and β7 mRNAs and their expression levels were significantly higher in WT BALB/c and IL-13 KO splenic K^d^Gag_197–205_-specific CD8^+^ T cells (in WT BALB/c and IL-13 KO groups, β1 expression, spleen vs. Peyer’s patch *p* = 0.001; β7 expression, spleen vs. Peyer’s patch *p* = 0.01) (Fig 8c & 8d). Furthermore, when the CCR9 and CCR10 mRNA expression were evaluated, very low numbers of gut- K^d^Gag_197–205_-specific CD8^+^ T cells (WT BALB/c, spleen 0/6 and Peyer’s patch 1/6; in IL-13KO spleen 1/5 and Peyer’s patch 3/9) were found to express CCR9 (Fig 8e) and no CCR10 mRNA expression was detected.

**Fig 9:**
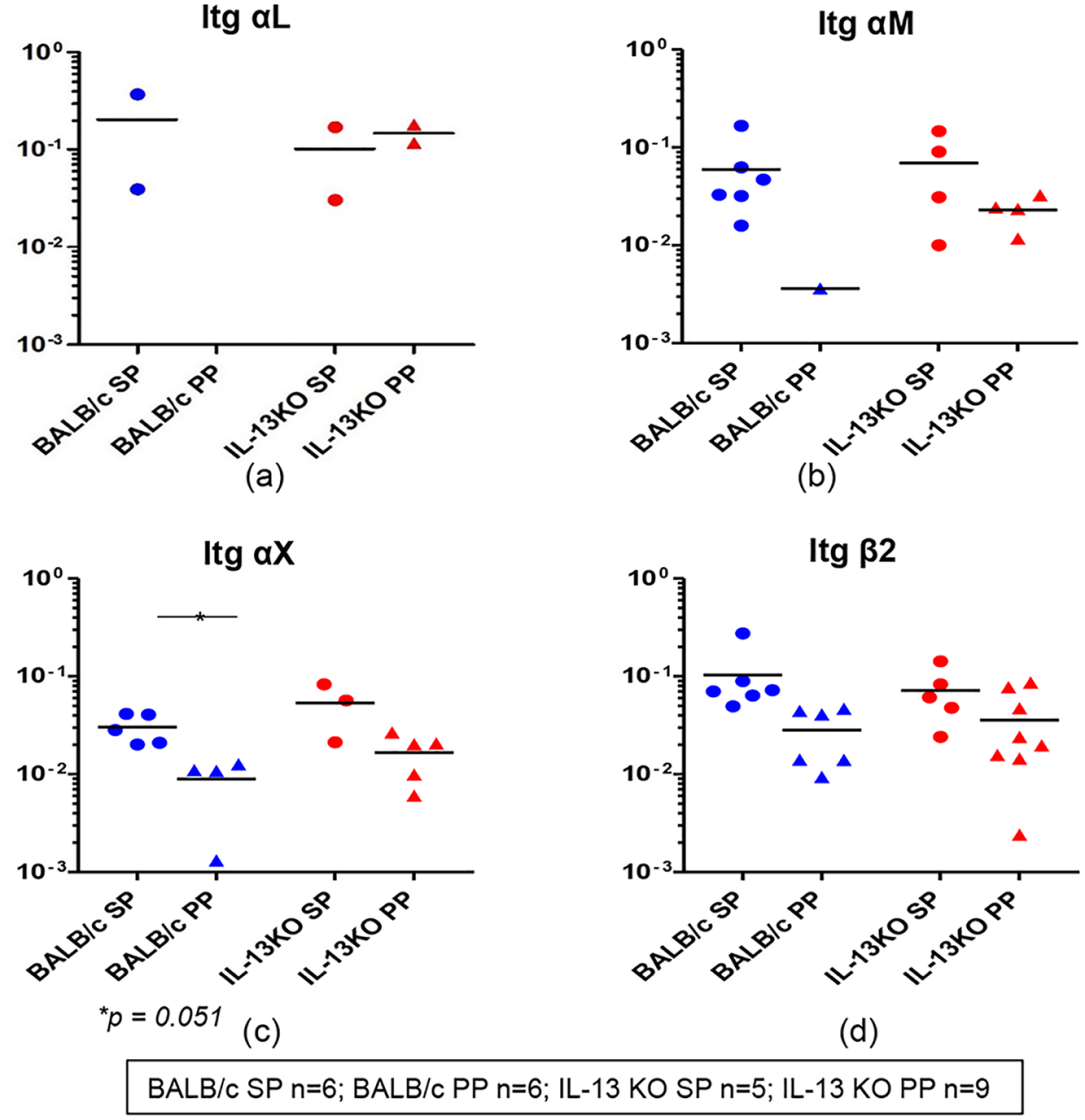
Evaluation of integrins αL, αM, αX and β2 mRNA expression in WT BALB/c and IL-13 KO gut and splenic K^d^Gag_197–205_–specific CD8^+^ T cells following i.n./i.m. vaccination. Exactly as in Fig 8, mRNA expression profiles in WT BALB/c and IL-13KO gut and splenic K^d^Gag_197–205-_specific CD8^+^ T cells were evaluated. WT BALB/c spleen (SP) and Peyer’s patch (PP) n = 6 pools of hundred K^d^Gag_197–205_–specific CD8^+^ T cells are indicated in blue circles and triangles respectively. IL-13 KO mice spleen n = 5 and Peyer’s patch n = 9 pools of hundred K^d^Gag_197–205_-specific CD8^+^ T cells are indicated by red circles and triangles respectively. Data indicate log mRNA expression of integrin αL (a) αM (b) αX (c) and β2 (d) normalised to housekeeping gene L32.

Furthermore, when the expression of integrin αL, αM, αX and αD mRNAs which are known to heterodimerize with integrin β2 were evaluated in both the groups, no integrin αD expression was detected. Lower proportion of gut/splenic K^d^Gag_197–205_-specific CD8^+^ T cells (20–30%) were found to express integrin αL and expression levels were similar in both the compartments and the two groups (Fig 9a). In contrast, higher numbers of WT BALB/c and IL-13 KO mice splenic K^d^Gag_197–205_-specific CD8^+^ T cell pools were positive for integrin αM compared to Peyer’s patch (WT BALB/c group, spleen 6/6 and Peyer’s patch 1/6; IL-13 KO group, spleen 1/5 and Peyer’s patch 4/9) and also the expression level was relatively elevated in splenic K^d^Gag_197–205_-specific CD8^+^ T cells (Fig 9b). Likewise, in all the groups tested, although the proportions of cell pools positive for integrin αX were found to be similar, expression level was higher in splenic K^d^Gag_197–205_-specific CD8^+^ T cells compared to Peyer’s patches (Fig 9c). Interestingly, most or all cell pools expressed obtained from both mice groups expressed integrin β2, and expression levels were relatively higher in splenic K^d^Gag_197–205_-specific CD8^+^ T cells compared to Peyer’s patch (Fig 9d).

### IL-13 KO mice have elevated α4β7^+^ and CCR9^+^ gut K^d^Gag_197–205_-specific CD8^+^ T cells

**Fig 10:**
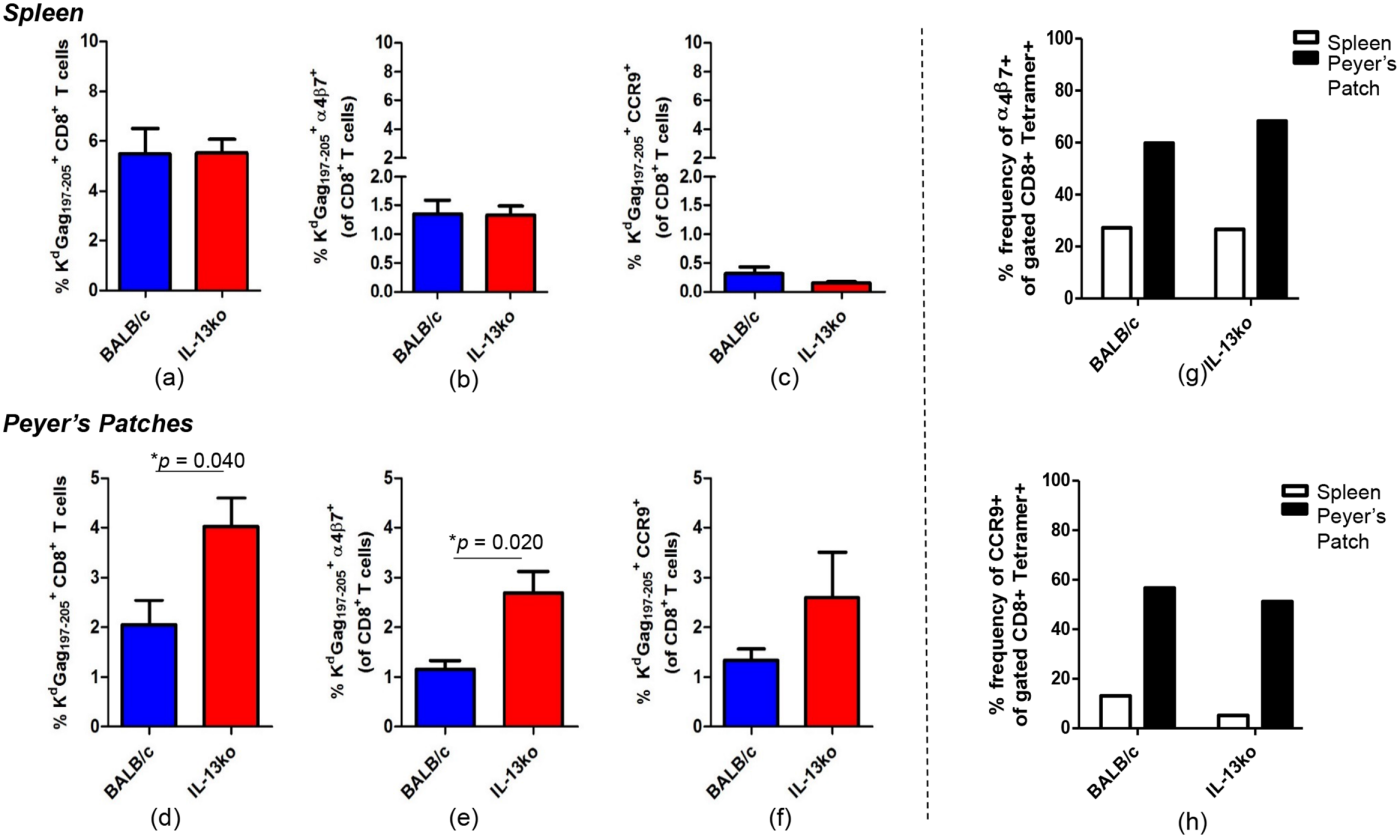
Evaluation of WT BALB/c and IL-13 KO gut and splenic K^d^Gag_197–205 –_specific CD8^+^ α4β7^+^ and K^d^Gag_197–205 –_specific CD8^+^ CCR9^+^ T cells following i.n./i.m. vaccination. WT BALB/c and IL-13 KO mice were prime boost immunized using i.n. FPV-HIV/i.m. VV-HIV strategy. Fourteen days post booster vaccination, splenic and gut K^d^Gag_197–205_-specific CD8^+^ T cell responses were evaluated using K^d^Gag_197–205_-tetramer staining together with α4β7 and CCR9 staining using FACS analysis. The percentage of WT BALB/c (blue) and IL-13 KO (red) splenic and gut K^d^Gag_197–205_-tetramer^+^ CD8^+^ T cells (a and d), K^d^Gag_197–205_-tetramer^+^ CD8^+^ α4β7^+^ T cells (b and e), K^d^Gag_197–205_-tetramer^+^ CD8^+^ CCR9^+^ T cells (c and f) is shown. The percentage frequency of α4β7 and CCR9 homing marker expression on WT BALB/c and IL-13 KO splenic (g) and gut (h) K^d^Gag_197–205_-tetramer^+^ CD8^+^ T cells is also shown. Data represent 4–5 experiments.

WT BALB/c and IL-13 KO mice were prime boost immunized using i.n. FPV-HIV/i.m. VV-HIV strategy. Fourteen days post booster vaccination, splenic and gut K^d^Gag_197–205_-specific CD8^+^ T cell responses were evaluated using K^d^Gag_197–205_-tetramer staining together with α4β7 and CCR9 staining using FACS analysis. Data revealed that the magnitude of K^d^Gag_197–205_-specific splenic CD8^+^ T cell numbers were similar between the two mice groups tested (Fig 10), which was consistent to our previous findings [12]. Notably, the percentages of α4β7^+^ splenic K^d^Gag_197–205_-specfic CD8^+^ T cells were much elevated in WT and IL-13 KO mice compared to CCR9 (α4β7^+^ ~1.5% and CCR9 less than 0.5%, *p* < 0.0001 (Fig 10b & 10c), but, no significant difference in expression was detected between WT BALB/c and IL-13 KO splenic groups (Fig 10b & 10c). In contrast, compared to WT mice the percentages of α4β7^+^ and CCR9^+^ gut-K^d^Gag_197–205_-specfic CD8^+^ T cells were found to be significantly elevated in IL-13 KO mice compared to the WT BALB/c (Fig 10e & 10f). When the frequency of α4β7 and CCR9 were assessed in spleen vs. gut K^d^Gag_197–205_-specific CD8^+^ T cells obtained from WT and IL-13 KO groups, threefold increase in the expression frequency of α4β7 and CCR9 were detected in Peyer’s patches (~ 60%) compared to spleen (~ 20%) (Fig 10g & 10h).

## Discussion

Studies have shown that mucosal immune responses are most efficiently induced by administration of vaccines to the mucosae, whereas systemic immunization strategies rarely induce long lasting or optimal mucosal immunity and are therefore less effective against infection at the mucosal surfaces [25, 26]. The Sabin live attenuated oral polio vaccine (OPV) introduced in 1950s is a classic example of an effective mucosal vaccine. Unlike the injectable inactivated polio vaccine (IPV), OPV was shown to induce secretory IgA antibody responses at the intestinal mucosae the site of virus replication helping effective control of poliovirus, making OPV a powerful strategy for global eradication of polio [27]. We have shown that i) vaccine regime (i.e. systemic vs. mucosal delivery) can significantly influence the magnitude as well as the quality/avidity of immune response at the mucosae [9, 10] and ii), mucosal verses systemic immunization regimes can induce distinct gene expression profiles in splenic K^d^Gag_197–205_-specific single CD8^+^ T cells [10]. In the current study evaluating the expression of thirty different genes in gut-K^d^Gag_197–205_-specific CD8^+^ T cells using Fluidigm 48.48 dynamic arrays have further established that different vaccine delivery routes can vastly influence the gene expression profiles in mucosal and systemic compartments. Specifically, compared to purely mucosal (i.n./i.n.) FPV-HIV/VV-HIV prime-boost immunization regime, combined systemic mucosal (i.n./i.m.) and purely systemic (i.m./i.m.) immunization regimes showed enhanced expression of MIP-1β, RANTES, and CCR5 mRNAs in gut K^d^Gag_197–205_-specific CD8^+^ T cells indicating that enhanced expression of these chemokines/chemokine receptor genes were most likely driven by the systemic antigen encounter following the i.m. VV-HIV booster vaccination. Our antibody array studies have also revealed enhanced expression of RANTES by HIV-specific CD8^+^ splenocytes following i.n. FPV-HIV/ i.m. VV-HIV immunization compared to i.m. pDNA-HIV/ i.n. FPV-HIV vaccination [6]. Furthermore, compared to WT BALB/c mice, IL-13 KO mice immunized with i.n. FPV-HIV/ i.m. VV-HIV vaccination strategy has also shown to induce higher avidity CD8^+^ T cells with better protective efficacy [12] and this was associated with significantly enhanced RANTES mRNA expression by splenic K^d^Gag_197–205_-specific single memory CD8^+^ T cells [12]. These studies also indicated that in antigen-specific CD8^+^ T cells, RANTES is regulated by IL-4 and lL-13 [12]. The current data further substantiated these findings as absence of IL-13 correlated with higher expression of RANTES and MIP-1β in splenic K^d^Gag_197–205_-specific CD8^+^ T cells.

In previous single cell profiling studies, majority of splenic K^d^Gag_197–205_-specific single (~80%) CD8^+^ T cells were found to express IFN-γ and lower proportion was found to express TNF-α (1–5%) [10]. However, in this study no IFN-γ expression was detected in the gut K^d^Gag_197–205_-specific CD8^+^ T cells. In contrast, in all three immunization regimes tested, 80–90% of the gut K^d^Gag_197–205_-specific CD8^+^ T cells were found to be positive for TNF-α. Interestingly, TNF-α have been associated with increased migration and proliferation of intraepithelial lymphocytes in gut [28] and this may explain the differences observed in CD8^+^ T cell cytokine specificities in spleen and gut compartments [6, 10]. Furthermore, the discrepancy in IFN-γ expression profile, compared to the previous studies [10] could also be due evaluating mRNA from 100 K^d^Gag_197–205_-specific CD8^+^ T cells not single cell level using Fluidigm 48.48 dynamic array analysis which could be a major caveat. Specifically, it is likely that in a pool of hundred cells the abundance of an mRNA like IFN-γ could be extremely high and this has high potential to inhibit optimum PCR amplification of these genes [29] [Note that in this study 30 genes were amplified from a single cDNA sample obtained from 100 cells and the abundance of the 30 gene transcripts will be vastly different (for example; elevated IFN-γ vs lower levels of integrin αD mRNA). Thus, in an ideal scenario the most abundant genes will have to be diluted to obtain the optimum amplification outcomes [29]].

Surprisingly, following i.n./i.m immunization no significant differences between the control WT BALB/c and IL-13 KO gut K^d^Gag_197–205_-specific CD8^+^ T cells were detected at a 100-cell level. However, previous studies in our laboratory have reported greatly elevated expression of granzyme B in IL-13 KO splenic K^d^Gag_197–205_-specific single CD8^+^ T cells compared to other groups tested [12]. Similarly, when mRNA expression profiles were evaluated at a single cell level enhanced expression of RANTES, MIP-1β, perforin and also granzymes A and B by gut K^d^Gag_197–205_-specific CD8^+^ T cells were detected in IL-13 KO mice compared to WT BALB/c mice (Trivedi *et*. *al*., manuscript submitted). Several other recent studies also have illustrated the power and necessity of using single-cell gene expression profiling approaches in evaluating vaccine efficacy or immune outcomes following infection [30–32]. For example, recent study by Flatz *et*. *al*., have shown qualitative differences between CD8^+^ T cell responses following different prime-boost immunization strategies (DNA/rAd5, rAd5/rAd5 and rAd5/rLCMV) when data were analysed at a single-cell gene expression level compared to pooled analysis [31]. Current data taken together with our single-cell gene expression data (Trivedi *et*. *al*., manuscript submitted) clearly reveal that when evaluating the outcome of an immune response, single cell gene expression profiling has high potential to generate more powerful information than pooled cell analysis.

Current study indicated that according to the anatomical location in which the K^d^Gag_197–205_-specific CD8^+^ T cells were obtained (spleen vs Peyer’s patch) the mRNA expression profiles were significantly different both in WT BALB/c and IL-13 KO mice. This is consistent with studies by Masopust *et*. *al*. [13] where they showed that anatomical location played an important role in modulating memory T cell immunity. Specifically, unlike Peyer’s patches, splenic K^d^Gag_197–205_-specific CD8^+^ T cells expressed CCR7 but lacked CD62L suggesting that tissue microenvironment regulated memory cell surface marker expression and memory T cell differentiation. Rapid down-regulation of CD62L by splenic K^d^Gag_197–205_-specific CD8^+^ T cells has often been correlated with the loss of T cell trafficking to peripheral lymph nodes [33]. According to the current findings it appears that gut K^d^Gag_197–205_-specific CD8^+^ T cells most likely retain CD62L expression which enable them to rapidly reenter lymph nodes [34, 35].

This study was one of the first to evaluate an array of integrins that are reported to be present on lymphocytes and their expression profile following different routes of vaccination and conditions. Several studies have now shown that integrins like α4β7 is differentially expressed on T cells and they play a role in tissue-specific T cell homing in an immunization route dependent manner [22, 24, 36–38]. Interestingly, current study clearly demonstrated that in a prime-boost vaccination setting, the prime or the booster immunization could differentially influence the integrin expression profile on gut K^d^Gag_197–205_-specific CD8^+^ T cells. For example; elevated numbers of integrin αE expressing K^d^Gag_197–205_-specific CD8^+^ T cells were detected in gut following mucosal but not systemic priming, indicating that the mucosal micro-environment was responsible for altering the αE homing marker expression. This is consistent with another finding where integrin β7 was also found to be up-regulated on T cells primed in mesenteric lymph nodes which home to the mucosae [37]. Integrin αE normally pairs up with β7 and is known to facilitate retention of lymphocytes in the gut via interaction with E-cadherin [39, 40]. In this study αE and β7 integrins were found to be more closely associated together in an i.n./i.n. vaccinated setting compared to the i.n./i.m or i.m./i.m. vaccinated groups. Recent studies have shown that during mucosal T cell priming, dendritic cells play a critical role in imprinting antigen-specific T cells with different homing markers and this is also retinoic acid dependent [20, 41]. Moreover, integrins α4 and β7 were found to be more closely associated together in all vaccinated groups tested compared to integrins α4 and β1. It is now well established that integrin heterodimer α4β7 help T cell migration to the gut mucosae [20, 22]. In the current study, following i.m. booster vaccination significantly enhanced integrin α4 mRNA expressing gut K^d^Gag_197–205_-specific effector CD8^+^ T cells were detected. This suggested that i.m. booster vaccination may have high potential to promote the expansion of mucosally originated antigen-specific CD8^+^ T cells (primed at the mucosae) in the systemic compartment. This hypothesis can be further substantiate as, higher α4 and β7 mRNA were detected also in splenic K^d^Gag_197–205_-specific CD8^+^ T cells suggesting that these cells may continue to circulate between the mucosal and systemic compartments. However, the full dynamics involved in tissue environment and the role of integrins in T cell homing is yet to be discovered.

Furthermore, following mucosal and systemic immunization all gut-K^d^Gag_197–205_-specific CD8^+^ T cells were found to express integrin β2. Studies have shown that integrin β2 (known to heterodimerize with αL, αM, αX and αD) play a critical role in T cell activation and in transendothelial migration [42, 43]. Unlike αL, αM, and αX, integrin β2 were found to be closely associated with integrin αD in an i.n./i.n. setting compared to the other two strategies tested. However, the function of integrin αD in a vaccination context has not yet been fully characterized. In contrast, the integrins αM and β2 were found to be relatively higher and closely associated in an i.n./i.m. and i.m./i.m. setting, further highlighting that immunization route/strategy can significantly regulate the expression of integrins on T cells.

Interestingly, compared to WT control the number of gut K^d^Gag_197–205_-specific CD8^+^ T cells expressing gut-homing markers α4β7 and CCR9 protein were found to be significantly higher in IL-13 KO as well as IL-4 KO mice (unpublished observation in our laboratory). New vaccines that transiently inhibited IL-4/IL-13 [44, 45] also enhanced integrin α4 mRNA expression in gut K^d^Gag_197–205_-specific single CD8^+^ T cells (Trivedi *et*. *al*., manuscript submitted). These observations suggest that inhibition of IL-13 or IL-4, most likely help increase trafficking of K^d^Gag_197–205_-specific CD8^+^ T cells to gut mucosae. Other studies also have shown the role of cytokines such as IL-12, IL-4 and TGF-β in regulating tissue-specific lymphocyte trafficking [46–48]. We have established that transient inhibition of IL-4 and IL-13 can significantly modulate the antigen presenting cell subsets at the vaccination site and induce a high avidity T cell repertoire [49]. Thus, we suggest that IL-4/ IL-13 depleted cell milieu most likely help create an unique T cell subset with specific homing capacities to the mucosal sites. The exact mechanisms how IL-4 and/or IL-13 modulate T cell homing warrants further investigation.

Collectively, the current data indicated that out of the thirty genes tested, at the pooled 100 cell level, the MIP-1β, RANTES, CCR5, perforin and integrin α4 mRNA expression were significantly different in the three immunization regimes tested. Similarly, the expression levels of MIP-1α, MIP-1β, RANTES, integrins α4, β1 and β7 mRNAs were markedly different between mucosal and systemic compartments. These observations further emphasize that the route of vaccine delivery, tissue microenvironment plus the cytokine milieu (specifically IL-13) can significantly influence the gene expression profiles of K^d^Gag_197–205_-specific CD8^+^ T cells and modulate their functional specificities (high verses low avidity) as well as cell homing capabilities. In conclusion, the above findings corroborate that these factors have to be seriously taken into consideration when designing novel/effective vaccines strategies that can induce optimal mucosal CD8^+^ T cell immunity against chronic pathogens such as HIV-1.

## Materials and Methods

### Immunization of mice

Pathogen-free 6 to 8 week old female WT BALB/c and IL-13 KO mice on BALB/c background were purchased from the Australian Phenomics Facility, the Australian National University (ANU). WT BALB/c mice were prime-boost immunized with 1 X 10^7^ PFU rFPV followed by 1 X 10^7^ PFU rVV expressing HIV-antigens, under isoflurane anesthesia 2 weeks apart using mucosal and systemic routes of vaccination. IL-13 KO mice were prime-boost immunized with i.n. rFPV/ i.m. rVV. The i.n. rFPV was given in a final volume of 20–25 μl, where i.m. rVV immunization was delivered in a 100 μl volume. All viruses were diluted in sterile phosphate-buffered saline (PBS) and then sonicated, to obtain homogeneous viral suspensions before delivery.

### Ethics Statement

All animals were maintained and experiments were performed in accordance with the Australian NHMRC guidelines within the Australian Code of Practice for the Care and Use of Animals for Scientific Purposes and in accordance with guidelines approved by the Australian National University Animal Experimentation and Ethics Committee (AEEC). This study was approved by the AEEC and listed under ANU ethics protocol number A2011/018. All animals were anesthetized using isoflurane and monitored daily. At the end of the experiments were ethically sacrificed using cervical dislocation in accordance with the above AEEC approved protocols.

### Mucosal and systemic lymphocytes preparation

The mice were euthanized using cervical dislocation, spleen and Peyer’s patches were removed and single cell suspensions were prepared as described previously [45, 50]. Briefly, the spleen cells were passed through a cell strainer and were treated with red blood cell lysis buffer for 7 min at room temperature, cells were then washed, and suspended in complete RPMI (RPMI supplemented with 7% fetal calf serum, 20 mM 4-(2-hydroxyethyl)-1-piperazineethanesulfonic acid, 30 μg ml^− 1^ penicillin, 50 μg ml^− 1^ streptomycin, 2 mM sodium pyruvate (Gibco-Invitrogen, Auckland, NZ), and 50 M 2-mercaptoethanol (Sigma, St Louis, MO), and kept at 4°C until use, similarly Peyer’s patches were prepared without the red cell lysis step. The Peyer’s patch cells were filtered through the small piece of gauze to remove debris prior to final suspension in complete RPMI medium.

### Tetramer and homing marker staining

Allophycocyanin (APC) conjugated K^d^Gag_197–205_ tetramers were synthesized at the Bio-Molecular Resource Facility at The John Curtin School of Medical Research (BRF/JCSMR), ANU. Tetramer staining was performed as described previously [9, 10]. Briefly, 2 X 10^6^ splenocytes and Peyer’s patches were stained with anti-CD8α FITC antibody and APC conjugated K^d^Gag_197–205_ tetramer at room temperature for 40 min. After washing cells twice with FACS buffer (PBS with 1% fetal calf serum), the cell pellets were resuspended in 0.5% PFA (except for studies involving cell sorting). For gene expression profile analysis, K^d^Gag_197–205_-specific hundred cells (from pooled splenocytes or Peyer’s patches) were sorted into 96-well plates using stringent gating strategy as described previously [10] [40]. NOTE: The purpose of this study was to assess the expression of these genes specifically in tetramer-specific CD8 T cells (with no prior antigen stimulation) similar to our previous studies. It is well established that antigen-activation (stimulation with specific-peptide) and tetramer staining cannot be performed concurrently, as i) stimulation prior to tetramer binding can lead to loss of tetramer staining due to down-regulation of the T-cell receptors and ii) peptide and tetramer competes for the same binding site and this leads to a competition and reduce tetramer staining.

For homing markers analysis, first the cells were stained with APC-conjugated tetramer for 40 min and then were surface stained with biotin labelled anti-mouse LPAM-1 or α4β7 and FITC conjugated anti-mouse CCR9 antibodies for 25 min at 4°C, followed by brilliant violet 421 streptavidin (useful for indirect staining of biotin labelled α4β7 primary antibody) for another 25 min at 4°C. Cells were fixed and total 10^6^ events per sample were collected using Fortessa flow cytometer (Becton-Dickinson), and the results were analyzed using Cell Quest Pro software. Note: frequency of homing marker expression out of the total K^d^Gag_197–205_-tetramer^+^ CD8^+^ T cell population was calculated as in Xi *et*. *al*. [50].

### Evaluation of mRNA expression in hundred K^d^Gag_197–205_-tetramer^+^ CD8^+^ T cells

K^d^Gag_197–205_ tetramer^+^ CD8^+^ hundred cells from spleen and/ Peyer’s patches were sorted using cell FACS Aria I (BD Biosciences) into 96-well plates containing CellsDirect qRT-PCR reaction buffer (Invitrogen), Platinum Taq polymerase / SuperScript III reverse transcriptase (Invitrogen), a mixture of Taqman primer-probes at 0.2X concentration specific for the transcripts of interest, as listed in Table 1 (Applied Biosystems), and SuperaseIn RNase inhibitor. Immediately following cell sorting, samples were centrifuged, and subjected to 20 cycles of pre amplification using polymerase chain reaction (PCR) (cell cycling as: 1 X 50°C 15 min for the reverse transcription then 1 X 95°C 2 min for reverse transcriptase inactivation and Taq polymerase activation, followed by 20 X 95°C 15 s and 60°C 4 min amplification cycle). Subsequently, the pre-amplified cDNA was stored at -20°C until analysis and was diluted with dH_2_O (1:2) prior to qPCR reaction. Each pre-amplified cDNA sample was then separated into 48 separate reactions for qPCR analysis using the BioMark 48.48 dynamic array nanofluidic chip (Fluidigm Inc., USA) according to manufacturer’s instruction.

Briefly, following hydraulic chip priming, 47 preamplified cDNA samples plus one no template standard were mixed with a mild detergent loading solution to allow capillary flow, and the samples were added to the sample inlets of 48.48 nanofluidic chip (Fluidigm, Inc.), 30 individual Taqman primer-probe mixtures (Applied Biosystems) specific for individual transcripts of interest listed in Table 1 along with assay loading solution were also added into the specific assay inlets of the 48.48 nanofluidic chip, allowing a combination of each sample to mix with each primer-probe assay in every possible combination (a total of 2304 reactions). The chip was then thermo-cycled through 40 cycles and Taqman primer-probe fluorescence in the FAM channel was detected using the CCD (charge-coupled device) camera attached to the BioMark HD system, normalized by ROX (6-carboxy-X-rhodamine) intensity. The statistical analyses of the data were performed as described below.

### Statistical analysis

Amplification signals were analysed and Ct values were determined using Fluidigm’s real-time PCR analysis software version 4. The Ct values of genes were normalized against housekeeping gene ribosomal protein L32 (ΔCt = Ct_gene_—Ct_L32_), data represents the mRNA fold expression relative to housekeeping gene (2^-ΔCt^). The error bars represents standard error of mean and the *p* values were determined using one-way analysis of variance followed by Sidak multiple comparison test or Dunnett (2-sided) post tests using IBM SPSS version 21. For the flow-cytometer analysis, the standard error of the mean and *p* values were determined using two-tailed Student’s *t*-test, using GraphPad Prism software version 5. Raw Fluidigm’s real-time PCR data can be found in supporting information (S1 and S2 Datasets).

## Supporting Information

S1 Dataset Ct values of genes expressed in gut HIV-specific CD8 T cells following different routes of immunization. (XLSX)

S2 Dataset Ct values of genes expressed in systemic and mucosal HIV-specifi CD8 T cells obtained from WT and IL-13 KO mice. (XLSX)
